# Bologna backstage – experiences from behind the scenes of the reforms of the Basel medical curriculum

**DOI:** 10.3205/zma001271

**Published:** 2019-10-15

**Authors:** Gabriele Voigt, Michael Wilde

**Affiliations:** 1 University of Basel, Faculty of Medicine, Office of the Dean of Studies, Basel, Switzerland

**Keywords:** reform efforts, topical blocks based on organ systems, hybrid curriculum, core studies and elective degree courses, general practice, european higher education reform, Bologna Declaration, resistance to change, structural implementation of the Bologna demands, cognitive affective psychomotor, longitudinal curricula, accreditation, PROFILES

## Abstract

Based on a six-year degree in medical studies which was characterized by a series of lectures without an over-arching concept, Basel decided to embark on reforms in 1995. The first wave of reforms (1998-2003) produced a hybrid curriculum structure with PbL teaching units, organized according to organ systems, in years 1-4 of medical studies, which met the demand for “clinical content in the pre-clinical phase”. A focus on General Practice medicine was achieved by implementing the “One to one tutorial” in the 3^rd^ and 4^th^ year of studies. Fixed weekly schedules provided space for instruction in the three learning dimensions – cognitive, affective and psychomotor learning – implemented in four longitudinal competence strands. The compulsory elective subject area “Early patient contact” was integrated into the elective degree courses of the Bachelor’s degree by creating projects.

From 2006 to 2012, the demands of the Bologna Declaration were implemented and a Bachelor’s and a “consecutive” Master’s degree program developed and then implemented. The PbL teaching units which had come in for criticism were replaced in the Master's Degree by the “Clinical Case” and in the Bachelor’s degree by the “Tutorial on Scientific and Clinical Reasoning”. To strengthen scientific competences, the “Science Month” and the compulsory elective subject area “Scientific Competences – Flexible Offers (WIKO.flex)” were introduced. Further curricular adjustments resulted in the development of an externally accredited integrated emergency curriculum, the establishment of feedback OSCEs and the intensification of the Skills Lab offer. In addition to content, organizational framework conditions for curricular development were key: Thus, the massive expansion of places on medical degree courses also had an impact on curriculum structure and examinations.

In 2017, PROFILES, the new Swiss competence framework, was published, which presents curriculum planning with new challenges as a result of the introduction of the EPA concept. Due to the flexible structure of the curriculum, the faculty feels confident it will be able to handle these challenges. The re-accreditation of the degree program in 2018 provided important stimuli for the strengthening of interprofessional teaching and a focus on the competent handling of an increasing number of patients asking for complementary medical treatments.

## Zusammenfassung

Ausgehend von einem sechsjährigen Medizinstudium, welches von diversen Magistralvorlesungen geprägt war, die sich konzeptionslos aneinander reihten, wurde in Basel 1995 beschlossen, eine Reform in Angriff zu nehmen. Die erste Reformwelle (1998-2003) brachte in den Studienjahren 1-4 eine nach Organsystemen geordnete hybride Curriculumsstruktur mit PbL-Lehreinheiten hervor, in denen der Forderung nach „Klinik in die Vorklinik“ entsprochen wurde. Eine Schwerpunktsetzung auf Hausarztmedizin gelang durch die Implementierung des “Einzeltutoriates“ im 3. und 4. Studienjahr. Festgelegte Wochenplanstrukturen schafften Raum für Unterrichte in den drei Lerndimensionen kognitiv, affektiv und psychomotorisch, umgesetzt in den vier longitudinalen Kompetenzsträngen. In das Mantelstudium des Bachelors wurde mit der Schaffung von Projekten der Wahlpflichtbereich gelegt, in dem der frühe Patientenkontakt umgestzt wurde.

Ab 2006 bis 2012 wurden die Forderungen der Bologna-Deklaration umgesetzt und ein Bachelor- und ein „konsekutives“ Masterstudium entwickelt und sukzessive implementiert. Dabei wurden in den Masterjahren die in die Kritik geratenen PbL-Lehreinheiten durch den „Klinischen Fall“, in den Bachelorjahren durch das „Tutorial of Scientific and Clinical Reasoning“ ersetzt. Zur Stärkung der wissenschaftlichen Kompetenz wurde der „Wissenschaftsmonat“ und der Wahlpflichtbereich „Wissenschaftliche Kompetenzen – flexible Angebote (WIKO.flex)“ eingeführt. Weitere curriculare Anpassungen führten zum Aufbau eines extern akkreditierten integrierten Notfall-Curriculums, zur Einrichtung von Feedback-OSCEs und Intensivierung des Skills-Lab-Angebotes. Neben inhaltlichen waren auch organisatorische Rahmenbedingungen für curriculare Entwicklungen ausschlaggebend: So hatte die massive Erweiterung der Medizinstudienplätze auch Folgen für die Curriculumsstruktur und Prüfungen.

2017 ist PROFILES, der neue Schweizer Kompetenzrahmen, erschienen, der mit der Vorgabe des EPA-Konzepts die Curriculumsplanung vor neue Aufgaben stellt. Aufgrund der flexiblen Struktur des Curriculums kann die Fakultät diesen Herausforderungen gelassen entgegensehen. Die Reakkreditierung des Studiengangs 2018 gab als wichtige Impulse die Stärkung von interprofessioneller Lehre und einen Fokus auf den kompetenten Umgang mit verstärkten Patientenwünschen nach komplementärmedizinischen Behandlungen auf den Weg.

## 20 years of Bologna reform set in context

First of all, the temporal context at the University of Basel should be emphasized: when the reforms of medical studies at Basel in the last 20 years are outlined in the following, it should be remembered this only represents a tiny part of the history of medical studies at Basel: In 2019 the faculty of medicine was able to look back on a 559-year history [1], [2]. The current claim of the University of Basel of “Educating talent since 1460” literally applies to the medical faculty: it was one of four founding faculties, with one full professor. Impressive names such as Paracelsus or Vesal are associated with Basel in the following centuries. When today’s students listen to their first lecture in the anatomy lecture hall, they do so a few meters away from Vesal’s 1543 skeleton which was prepared in Basel. The history of medicine in Basel is also kept alive through the collections of the anatomical museum, the historical museum and the museum of pharmacy. In view of the long tradition, the Bologna reforms, which have been seen as revolutionary by some, seem like a rather small change, one of many that have been taken in the past and without doubt also in the future. 

## The will for “gentle” Bologna reforms in Basel

The faculty of medicine in Basel had already decided on a “gentle reform” in early 1995 because it was no longer able to escape criticism of the hitherto very traditional “talk and chalk” curriculum. In the pre-Bologna era (1995-97/016), one reads of a “certain malaise in the classroom” in faculty protocols.

In 1995 a working group drafted a rough concept for a reform of clinical teaching on the basis of the Commission reports of the Swiss Medical Interfaculty Commission (SMIFK) and a series published in 1995 in the Lancet on study reform. Several key elements were defined: reduction of lectures by 20%, with continual rise in teaching staff, reduction of anonymity, introduction of a subject matter catalog, introduction of tutorials in which case histories are dealt with, as well as the introduction of a 6-week internship at a hospital of the University of Basel. The formation of an institute for study organization and planning was deemed essential for this project, with a half-time professor and secretariat. The faculty approved the application and instructed the dean of studies to implement this reform. The faculty protocol (1995-97/056) states: “The faculty of medicine notes that such a reform should be gradual and gentle, based on existing structures and understood as a long-term process.” 

The reformers had to face several major challenges: There was no learning catalog at all, teaching staff were not always supportive and the spatial infrastructure and staffing for such a reform project was in short supply. The then director of the “reform bureau” which had been set up only had a budget of CHF 350,000 for a period of two years and had virtually no classrooms for small group teaching.

A large pharmaceutical company from Basel came to the rescue and provided a building in which the small group teaching and the new OSCEs could be carried out. However, this teaching building – referred to as the “brainbox” – was located in the Basel harbor area, which was not ideal, and was not integrated into the adjacent campus area which was otherwise contiguous. This circumstance had a significant impact on the acceptance of the PbL tutorials taught in this building.

In addition to a doctor, an educationalist was hired by the reform bureau. Using the specifications of the faculty and the old reform ideas of Habeck, Schagen and Wagner [3] and the recommendations of the Murrhardt Circle [4] and publications such as Tomorrow’s Doctors [5], they developed a didactic concept based on the Maastricht model PbL curriculum. Specifically, this meant implementing a degree course split into core (compulsory) subjects and elective degree courses. The core studies were designed as integrated thematic blocks in which organ-specific emphases enable the integration of subjects, alongside problem-oriented cases and practical courses. This was done in order to implement all three training dimensions – knowledge, skills, attitudes – into teaching and examinations. The elective subject area was to be covered by theme days and projects. Using the idea of the teaching-learning spiral, thematic blocks with the same system relations were designed, starting with the 3^rd^ and 4^th^ year (the so-called clinical phase) and then the 1^st^ and 2^nd^ year of the course (the pre-clinical phase). Practical teaching at the bedside was introduced under the label “doctor patient lessons” in the 3^rd^ and 4^th^ year of the course and tested through a 6-station OSCE. general practice was strengthened by the introduction of one to one training with a GP: In this tutorial, every student in the 3^rd^ and 4^th^ year would attend a working GP surgery once a week for half a day. For the purpose of quality assurance, student feedback providers – so-called contact groups – were set up throughout the core curriculum. They gave personal and structured feedback on a thematic block to every thematic block leader in the presence of a representative of the office of the dean of studies.

## Law on medical professions, the Fleiner commission and pilot accreditation

In 1998 there is a reference in the protocol of the faculty meeting of the faculty of medicine of Basel on the establishment of a federal commission of experts under the direction of Mr Fleiner, a constitutional lawyer. This Fleiner commission redesigned the law on medical professions and replaced the term state examination with the more correct – in a Swiss context – legal term *federal examination*, which became part of the law. The aim was the deregulation of medical studies, which should have led to a transfer of competences to the universities, for example with regard to authorization requirements of partial examinations or central validation of grades. 

The accreditation of the pilot following the reforms did confirm that the faculty’s efforts were worthy of praise but that the degree course was too lecture-intensive, that “aims and objectives” were lacking, that the first two years had to be restructured, and that “some lecturers still had not come on board with the study reforms.” The faculty in Basel perceived the whole process as a burden. According to the faculty’s protocol (1998-2000/078) notes that the president of the internal accreditation commission reported to the Faculty “that the climate in the commission was perceived as somewhat aggressive and pointed”. 

In addition, the reform bureau had begun implementation of the thematic block structure in the 3^rd^ year (5^th^ semester) and continued with it in the 4^th^ year (7^th^ semester) because more support from the hospitals for an organ-/system-based, thematic block-structured curriculum was hoped for. Following a rebuke from the accreditation commission, the thematic block structure was implemented in the first two years of study, the first in 2001/02 and the second in 2002/03.

**Figure 1:**
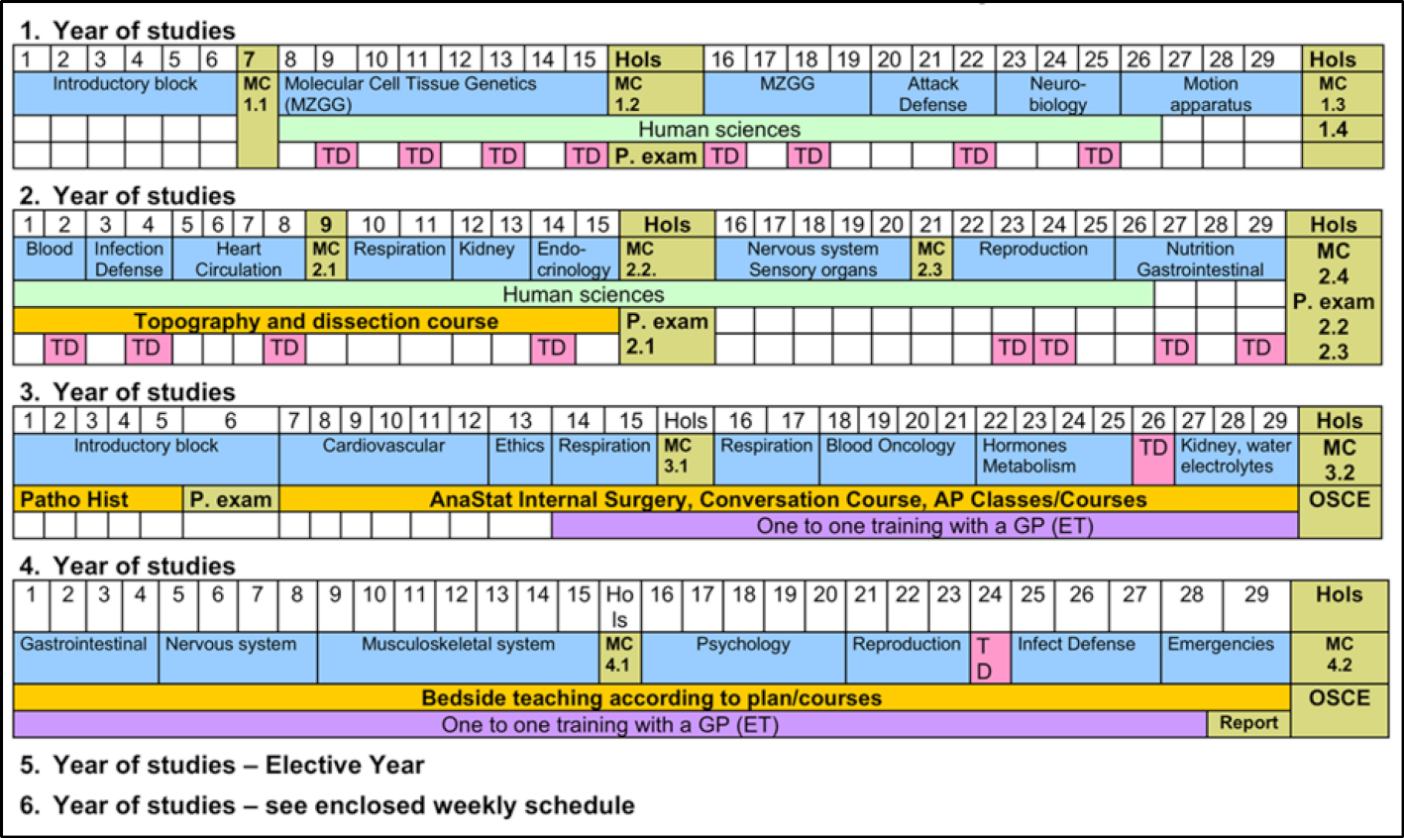
Basel curriculum in human medicine in the academic year 2002/03

These thematic blocks too were planned by integrating subjects based on organ systems, with practical references and PbL teaching units (see figure 1 (Fig. 1)). The result was a hybrid curriculum because completely abandoning the talk and chalk elements was politically unenforceable. This meant that the two weekly PbL units were not the central curricular units which could then be supplemented with orientation lectures but rather the lectures were the central elements of the curriculum for teaching content and the PbL units served to “integrate knowledge”. For this reason these lessons were christened Problem-oriented Tutorials (PoT) in Basel.

## From hybrid curriculum to competence strands

As the cohorts from the reformed first and second year moved into the 3^rd^ year, an increasing number of redundancies were found because the integration of subjects and the desire for “clinical content in the pre-clinical phase” in the first two years covered much of the content of the following years. So the thematic blocks of the 3^rd^ and 4^th^ year had to be completely revised again. The project concept, which was developed in Münster with the aim of early patient contact, was offered as an elective course in the first year of medical studies in 2003/04. The so-called “learning on a project” was offered as a choice and was designed as a small group course. It was performed with tutor support and had strong clinical references.

The evaluations of the thematic blocks in all 4 annual courses between 2003 and 2005 brought a very heavy lecture load to light. Many thematic block leaders scheduled lectures in the time slots that were actually intended for practical teaching. In a departure from the thematic block logic, all practical lessons relevant to being a doctor were identified and categorized. Thus 4 longitudinal curricula of basic competencies were developed: scientific competences; manual, diagnostic and therapeutic skills; social communicative and ethical competences; and humanities. These lessons were gradually implemented as thematic block-independent longitudinal curricula into their designated time-slots in the weekly schedule. 

Based on many evaluation results, which were not always satisfactory and many subsequent improvements and necessary adjustments, a certain amount of reform fatigue set in. From the point of view of many subject representatives, the work of the reform bureau was primarily perceived as taking away teaching time. Additionally encouraged by the critical report of the accreditation commission and faculty internal tensions, acceptance for the reforms dropped to a minimum. The reform bureau was subject to strong opposition, culminating in a “resistance to change” when the “gentle Bologna reform” which had initially been intended degenerated into a reform monster. 

## Harsh pressure

The pan-european university reform movement did not shy away from the University of Basel either. In June 1999, the Bologna declaration was adopted in europe and also signed by Switzerland. The then rector in Basel was a firm advocate of the european higher education area and pushed the reforms at the University of Basel. He argued that the faculty of medicine should not remain an unreformed island within the university. In his view, Bologna offered an “opportunity for medicine” [6], and with the Swiss rectors’ conference putting down their foot, there was no more room for doubt. There would be “no special route for medicine!”, there was no desire to fall out of line with the alma mater, as suggested by the expert group of secretary of state Kleiber [7]. 

In the faculty, this pressure left its traces. “reform” was considered a dirty word by some and didactics became a red rag. Although seen from a distance an incredible amount had been achieved, the atmosphere between the office of the dean of studies which had by now been set up, the former reform bureau, and teaching staff of the clinical and core subjects had turned icy. Open resistance manifested itself in the lecture theaters with lecturers regularly starting lectures by stating that they were no longer able to teach everything and that the students would end up with gaps in their knowledge because the 0ffice of the dean of studies had canceled the necessary hours. Only the powerful incumbent dean was able to force the responsible thematic block leaders of the core and clinical subjects to comply with the draft for the Bologna structure. He called each thematic block leader into his office and made them sign an agreement that the thematic block leaders committed to adhere to.

The draft for the new Bologna curriculum aimed to retain as much as possible of the reformed curriculum which by now covered years 1 to 4. This turned out to be quite difficult because in view of the impending Bologna reform nothing had been changed after the 4^th^ year, leading to a situation where two differently structured phases had to be merged into a single new concept. The 5^th^ year of medical studies had remained as the Elective Year (similar to the Practical Year) and the 6^th^ year continued to include subjects such as internal medicine, surgery, neurology and gynecology, taught as talk and chalk lectures and which now had to be integrated into a uniform Bologna structure. The subject representatives clung to their traditional structures, so the elective year had to be postponed by one semester to make room for the subjects formerly predominantly taught after the elective year. Every single hour was counted to determine the proportions of the subjects to each other and to plan the dimensions of the new thematic blocks in the same ratios. Although the whole process was characterized by great fear of loss, there were also upsides. It was finally possible to set up thematic blocks for certain subjects which had no (or too little) presence in the old structures before the practical year, such as legal medicine, ENT, ophthalmology, reproduction, life cycles, evidence-based medicine, boundary areas, and their like.

In addition, a favorable starting position had resulted: the newly established thematic block structure enabled a meaningful horizontal and vertical linking of teaching content, project work provided the concept and content for the electives (elective degree courses) and the longitudinal curricula of the basic competences fulfilled the demands of the new law on medical professions. The office of the dean of studies was granted insight into a pre-release version of the planned law and it became clear that the plans in Basel were in line with the new law.

## The Bologna model as a starting point

Bologna consists of a three-level model, in which the bachelor’s degree takes 3 years, the master’s between 2 and 3 years; and Doctoral studies another 3 years. The guidelines stipulated that the bachelor’s degree should provide the basic training and the master’s degree in-depth training. The reform had just recently introduced state-of-the-art integrated thematic blocks in Basel which best met the demands for “clinical content in the pre-clinical phase”. There were frenzied discussions about whether everything would have to be reversed but a decision was made not to. Bologna had to be implemented but the innovations in our curriculum were to be preserved. In order to meet the calls for a split into a bachelor’s and master’s degree, the didactic concept of the learning spiral was put to use. In the bachelor’s degree, the organ-related thematic blocks with special emphasis on anatomy, physiology, pathology and pathophysiology are taught using clinical examples. In the master’s degree, students go through the same organ-based thematic blocks with special consideration for clinical topics, diagnostics, treatment and differential diagnoses. 

Training in the four basic competences was to result in the “advanced competencies” in the master’s degree. Projects, which offer choice to students, were continued until the 3^rd^ year of the bachelor’s degree. For reasons of capacity, the one to one training with a GP, which had been introduced so successfully, was made an elective degree course in the 1^st^ year of the master’s degree.

## Dead end: major tracks

Bologna’s call for early specialization was a particular challenge. Four different “majors” were developed, each leading to a different degree: human medicine to a master of clinical medicine, dentistry to a master of dental medicine, biomedical sciences to a master of biomedical science and public health to a master of public health. These four tracks were filled with students based on their school leaving grades, with 10 students each in the majors biomedical science and public health, 40 in dentistry and about 120 in human medicine. In the first year of the bachelor’s degree, projects in these four directions were offered and a curricular focus was developed for the two following years. 

However, when the students interested in public health and biomedical science reached the third year of the bachelor’s degree with the majors to be continued in the master’s degree program by specialization, the students found this blocked their route to the federal examinations and to becoming clinical doctors. The responsible lecturers advised against it and the students refused to continue on this path because they had explicitly opted for a clinical career when choosing their studies. The lesson learned was that students of human medicine selected by numerus clausus were not the right target group for this direction of specialization. A completely separate application procedure, possibly with a different numerus clausus, would have had to be conducted in order to attract students likely to succeed for these majors. But there was no appetite for this extra effort, so the two majors were not continued in the master’s degree. However, much of the teaching content has been carried over into the teaching of basic and advanced competences.

## Personnel implementation of the reforms

**Figure 2:**
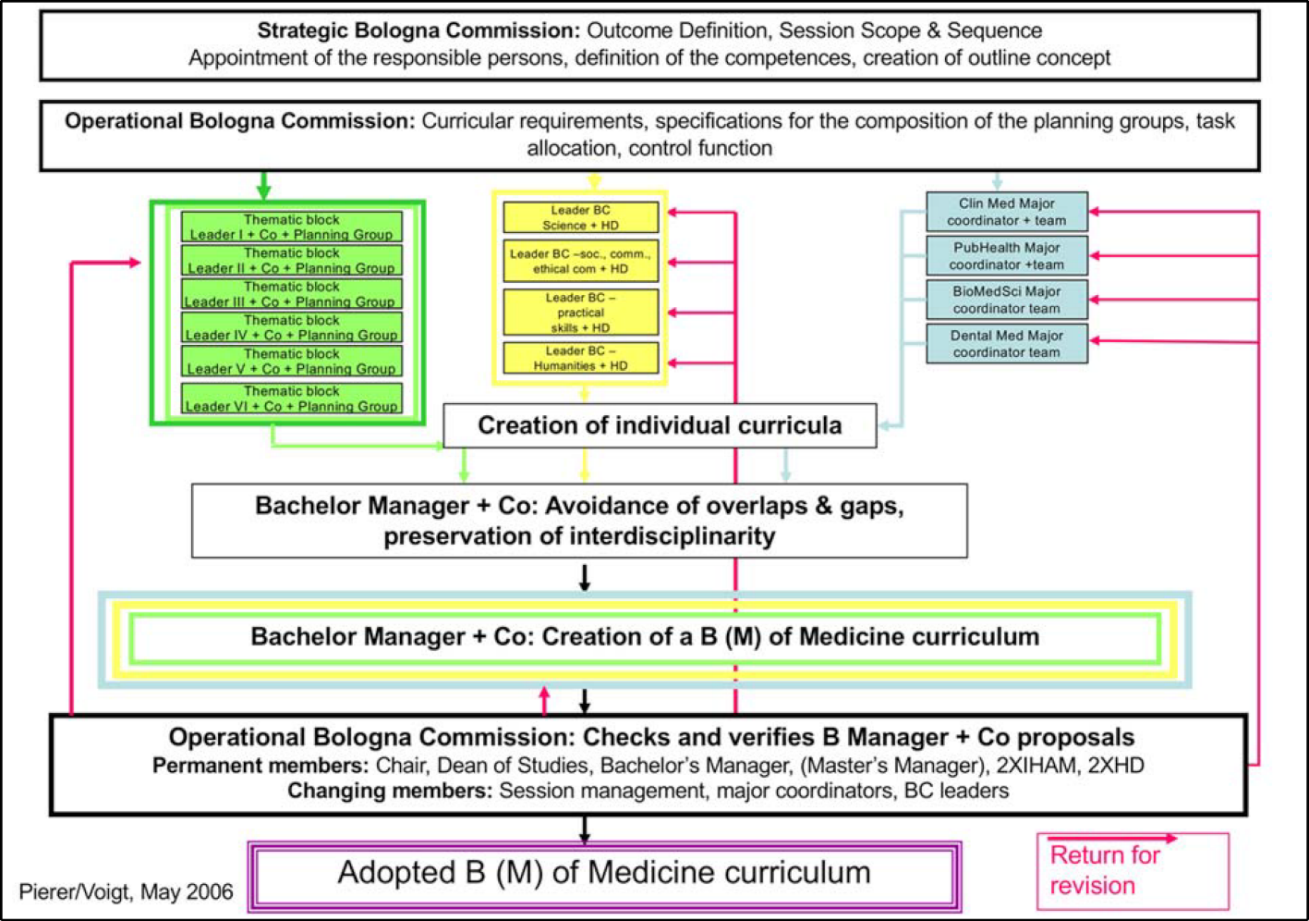
Diagram of the curriculum planning processes after the Bologna reforms

The positions of bachelor’s and master’s manager were created, a Bologna Commission set up and people responsible for the majors and the basic/advanced competences nominated. Figure 2 (Fig. 2) shows how complex this setup was. Then the staff of the office of the dean of studies combined a lot of dedication, expertise and extremely good links to the hospital with the necessary assertiveness of the Dean and thus made it possible to bring the faculty along. Each academic year, each thematic block, each major and each basic/advanced competency was examined for horizontal and vertical linking after preparatory work in the planning groups and by the bachelor’s and master’s managers in the Bologna commission and passed on to the curriculum commission for approval. This was a herculean task and the protagonists of the time agree that the favorable, motivating group of people in the office of the dean of studies had generated the thrust needed to bring this task to fruition. In the accreditation report 2011, the reform efforts and the compliance achieved with the regulatory requirements were assessed positively. The recommendation for accreditation was made without conditions. 

## From problem-based learning (PbL) to clinical cases and tutorials of scientific and clinical reasoning (TSCR)

For many years, the PbL teaching format in Basel had been criticized by students and lecturers. The students did not see the point of brainstorming about their knowledge gaps, as required in the PbL. And the lecturers felt out of place, just sitting there and not being allowed to say anything. Both sides felt demotivated, especially since the teaching format was mainly carried out in the decentralized brainbox – the rejection of the teaching site was associated with the rejection of the teaching format. Even intensive development efforts by the faculty could not prevent this. 

When Bologna was introduced, it was decided that in the master’s phase the small group format should in future be carried out as a case presentation in the presence of a tutor as a so-called clinical case, based on the principles of progressive disclosure. In the bachelor’s degree, after the third critical submission by the students in the faculty meeting in 2014, it was decided to commission the faculty's didactic experts with the development of a new format. With the help of the students the “Tutorial of Scientific and Clinical Reasoning (TSCR)” was developed based on the flipped classroom model, which provides for preparation, an examination of what has been learned, moderation by a tutor and working through a case in a classroom setting. This concept was successively introduced in the bachelor years from 2015/16, replacing the earlier PbL in the Basel curriculum. The two new formats meet the needs of students and lecturers in every respect and the teaching compliance has improved noticeably, something that is clearly reflected in the evaluation results [8].

## Examinations

Already with the first implementation of bedside teaching in the 3^rd^ year of medical studies in 1998/99, a 6-station OSCE was introduced and then implemented in the following 4^th^ year. It remained in place until 2001/02, when following a change of personnel neither the cases, the planning documents nor the standardized patient file could be traced. In 2002/03 things had to be started again from scratch.

Since then, the OSCE implementation has repeatedly been updated. Initially, some skills were tested in the 1^st^ year, followed by the introduction of evaluation competences in the OSCE by working with images and then anatomy tag test was added to the OSCE examination. Later the assessment competences were integrated into the digital mc exams and from then onwards only practical and communicative skills were examined again in the OSCEs. 

With the introduction of the federal examination in 2011, the examination load in the office of the dean of studies became unmanageable and ways of streamlining examinations were considered. Currently (2019) a formative and a summative OSCE are conducted in Basel, both with feedback, in the 3^rd^ year of the bachelor’s degree. In the 2^nd^ year of the master’s degree, before entering the elective year there is a summative 12-item OSCE with digital feedback and in the 3^rd^ year of the Master’s degree before the federal exam a formative 6-station OSCE with electronic feedback. In all years, a summative MCQ examination takes place after each semester. The projects are completed with a portfolio examination and the basic competencies are formatively examined during the academic year without an OSCE using a course certificate report card. During the elective year a logbook has to be kept and now the introduction of Mini-CEX and DOPS is being piloted in bedside teaching. 

## Further innovations after 2010

A curriculum is never static but always in the process of being updated. Student and graduate feedback in the contact group sessions is constantly used as an opportunity to introduce further innovations. Thus, in 2011, the “science month” (WIMO) was introduced before the elective year. It consists of a simulated congress, with students mutually assessing the abstracts of their Master’s theses and receiving feedback from tutors. The best then present to the full cohort. In 2016 a further adjustment of the scientific strand was made. Statistical content that had been introduced too early in the second year was moved to a longitudinal compulsory elective subject area. In addition to the newly developed statistics e-tutorials, this compulsory elective subject area was also expanded to include additional content and formats, including journal clubs, university library offers and a basic course in good clinical practice. Within scientific competence a sub-strand was thus created between the 3^rd^ and 5^th^ year with flexible offers (WIKO.flex), from which the students can choose those that fit their master’s thesis and at a time of their choosing. In a further innovation, WIKO.flex is fully controlled digitally via the learning management system.

Medical emergency training was scrutinized early on and it was determined that there was only one practical unit missing to meet the requirements for the duty doctor course of the Swiss Society for Rescue and Emergency Medicine (SGNOR). Following the introduction of this medical emergency training station, the faculty has been accredited for medical emergency training and has since been able to issue an equivalent to the duty doctor course certificate required for various medical specializations. Training in palliative care has also been intensified so that instruction now meets the requirements of the European Association of Palliative Care (EAPC).

The introduction of formative OSCEs and feedback about it showed that practical training was still seen as deficient. The doctor patient classes in hospitals could not be expanded for capacity reasons. To strengthen practical teaching, therefore, the skills lab was expanded and “practicing under guidance” introduced. In the bachelor years, the participants of the practical skills training are instructed by student tutors, in the master years by clinical tutors. The need for tutors increased and the need to teach them the “Basel standard” of skills suggested a need for standardized training. Together with the requirements in the postdoctoral lecture qualification process, in which university didactic training is demanded of the candidates, this led to a hitherto dormant medical didactics program being reanimated and broadened to an extended faculty development program.

## Switzerland-wide challenges: rising student numbers and PROFILES

Due to political demands and labor market conditions, the offering of places to study medicine in Switzerland has grown increasingly dynamic since 2010: capacities will be significantly expanded at existing sites and in cooperation with existing universities, additional universities will offer sections of medical studies, which will inevitably result in intra-swiss mobility and course credit transfer (for example, bachelor studies at the new ETH site in Zurich, ,aster’s degree in Basel). The integration of additional students is taking place in waves over a decade (2014-2024) and in Basel will result in almost doubling the number of places. Such an increase means massive additional organizational and planning burdens and will inevitably have consequences for curriculum planning. One consequence already mentioned was the reduction of summative OSCE examinations, which was compensated by formative elements.

Yet another change to the framework conditions will be the replacement of the previous Swiss Learning Objectives Catalog (SCLO [9]) with the learning target framework PROFILES (Principal Relevant Objectives and a Framework for Integrative Learning and Education in Switzerland [http://www.profilesmed.ch]). From 2020 onwards PROFILES will form the basis for the federal examination and will also be based on the concept of Entrustable Professional Activities (EPAs) in addition to the CanMEDs roles already familiar from the SCLO and the problem-based learning approaches. Basel’s medical curriculum in principle is well set up as an integrated and competence-based curriculum with a view to the specifications of PROFILES. Also, the coverage of PROFILES learning targets by the existing curriculum is not bad overall. Future efforts will be devoted to identifying and closing gaps in this context. Likewise, the development of clinical instruction with patients and their on-site assessment (e.g. Mini-CEX) remains a central task of curriculum development.

## The state of affairs in 2019 and outlook

**Figure 3:**
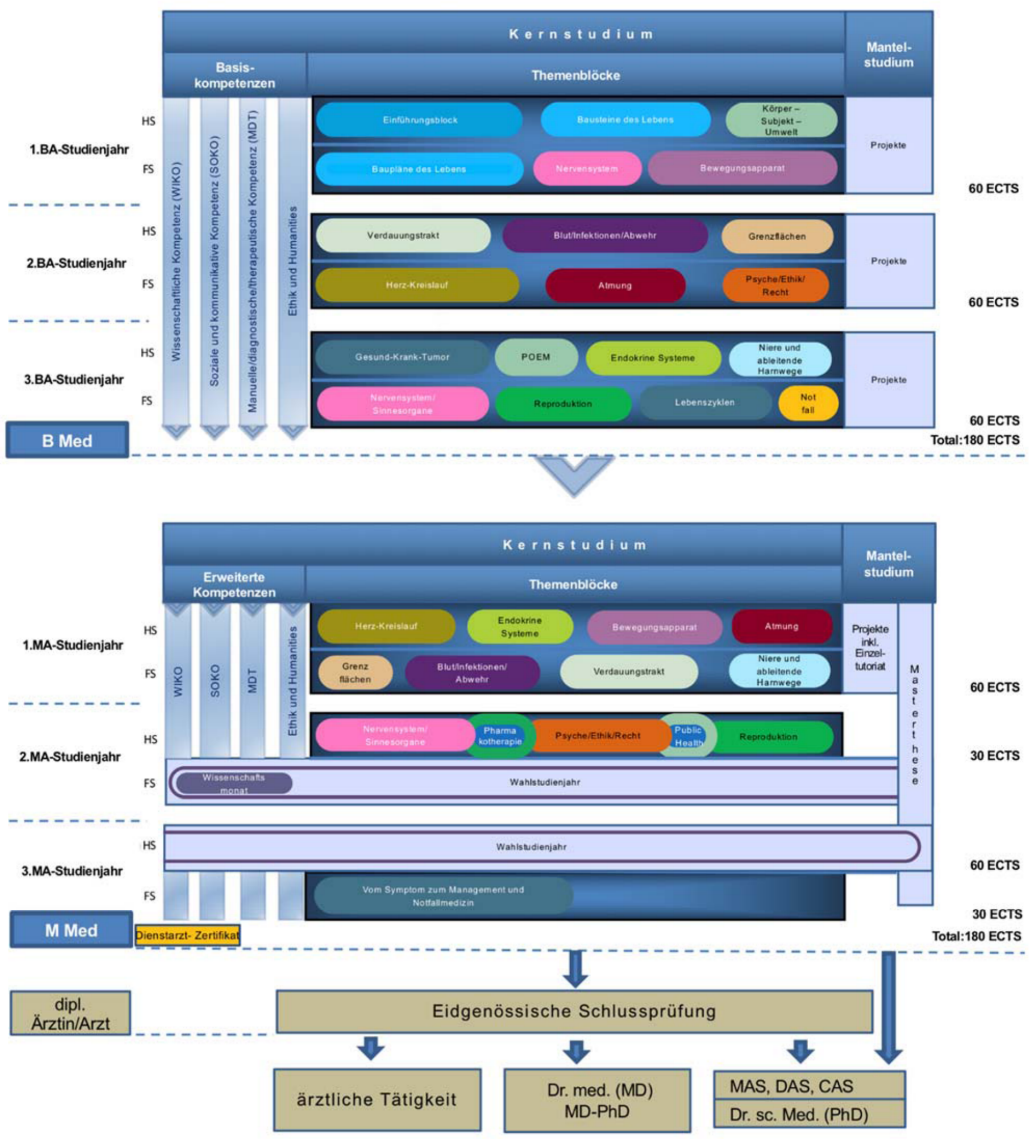
Human medicine curriculum (as of 2019)

The current state of the Basel curriculum is shown in figure 3 (Fig. 3). Of course this will soon be out of date again as medicine does not stand still and the societal expectations of future physicians change, so curriculum development must take this into account. With the expansion of interprofessional teaching and a stronger emphasis on (critically classified) complementary medicine, the reaccreditation commission gave important stimuli for further development in 2018 but the list of reform projects could still be expanded by: extending practical sonography lessons, expanding medical mindfulness and career planning or strengthening blended learning formats are just some of the topics currently being worked on.

## Competing interests

The authors declare that they have no competing interests.
